# Exercise Participation and Rehabilitation in Cardiomyopathies: An Updated Review

**DOI:** 10.3390/jcm14248683

**Published:** 2025-12-08

**Authors:** Domitilla Russo, Cristina Gambardella, Maurizio Volterrani, Rosa Lillo, Massimo Volpe, Camillo Autore

**Affiliations:** 1 Department of Cardiology, Santo Spirito Hospital, 00193 Rome, Italy; russo.domitilla@gmail.com; 2 Unità di Riabilitazione Cardio-Respiratoria, IRCCS San Raffaele Cassino, 03043 Cassino, Italy; cristina.gambardella@gmail.com; 3 IRCSS San Raffaele Roma, Università San Raffaele-Uniroma 5, 00166 Rome, Italy; maurizio.volterrrani@sanraffaele.it; 4 Department of Cardiovascular Sciences-Heart, Fondazione Policlinico Universitario A. Gemelli IRCCS, 00168 Rome, Italy; lillo.rossa@gmail.com; 5 IRCSS San Raffaele Roma, Via di Val Cannuta 250, 00166 Rome, Italy; massimo.volpe@uniroma1.it; 6 Department of Cardiology and Respiratory Sciences, IRCCS San Raffaele Cassino, Via G. di Biasio, 1, 03043 Cassino, Italy

**Keywords:** cardiomyopathy, hypertrophic cardiomyopathy, dilated cardiomyopathy, arrhythmogenic cardiomyopathy, cardiac rehabilitation, exercise, sudden cardiac death

## Abstract

Cardiomyopathies, including hypertrophic (HCM), dilated (DCM), and arrhythmogenic (ACM) forms, represent a challenge in cardiovascular medicine, in particular regarding exercise participation and cardiac rehabilitation. Traditionally, physical activity was restricted in these patients due to concerns over arrhythmic risk and sudden cardiac death. However, current evidence suggests that individualized exercise programs, under clinical supervision, can enhance functional capacity, improve quality of life, and sometimes prognosis in selected patients. Contemporary European and North American guidelines suggest that participation in competitive sports may be reasonable for athletes with genetic cardiomyopathies, provided that individual risk is regularly and systematically reassessed. The aim of this review is to synthetize current evidence on exercise training, sports participation and rehabilitation in the three major cardiomyopathies—hypertrophic, dilated, and arrhythmogenic—which have informed the latest international guideline recommendations. Particular attention is given to the essential role of shared decision-making, highlighting the importance of a personalized approach based on the specific type of cardiomyopathy, arrhythmic risk stratification, and individual patient factors. In addition, the review addresses two emerging clinical scenarios: sports participation in patients with implantable cardioverter-defibrillators and current recommendations for genotype-positive/phenotype-negative individuals at risk of cardiomyopathy.

## 1. Introduction

Regular physical activity and systematic exercise confer several cardiovascular, psychological, and quality of life benefits. Cardiovascular disease is the leading cause of global morbidity and mortality, and low levels of physical activity is a leading independent predictor of poor cardiovascular health [1]. In recent decades, the interest in the role of sport, physical activity, and cardiac rehabilitation in patients with cardiomyopathies has grown both in clinical practice and in scientific literature [2]. Exercise, when appropriately prescribed, is a cornerstone of cardiovascular health, and the benefits of regular physical activity have been consistently demonstrated. It reduces atherosclerotic burden and lowers the risk of coronary artery disease, enhances hemodynamic and metabolic profiles by improving blood pressure and lipid control, and contributes to the prevention of obesity [3]. Individuals who exercise regularly live approximately six years longer than sedentary individuals and exhibit a reduced risk of cerebrovascular events and certain malignancies [4]. Physical activity also has a favorable impact on mood, alleviating anxiety and depression and thereby supporting both physical and psychological well-being [5]. Furthermore, exercise promotes endothelial health, optimizes autonomic balance, and increases peak oxygen uptake (VO_2_ peak), all of which are key prognostic markers for cardiovascular and all-cause mortality [3]. Physical activity, and especially sports participation, has traditionally been discouraged and, in some cases, contraindicated in specific forms of heart disease such as cardiomyopathies. Furthermore, rehabilitative interventions have not been considered for the clinical manifestations of heart failure complicating these conditions [6]. Indeed, prescribing physical activity in patients with cardiomyopathies—i.e., with hypertrophic cardiomyopathy (HCM), arrhythmogenic cardiomyopathy (ACM), and dilated cardiomyopathy (DCM)—represents a clinical challenge. Cardiomyopathies are the leading cause of exercise-related sudden cardiac death (SCD) in young people in the Western world [7,8,9]. Cardiomyopathies are characterized by profound structural and functional myocardial alterations that predispose patients to an increased risk of arrhythmic events, progressive heart failure, and sudden cardiac death, particularly under the stress of high-intensity exercise. In the presence of the underlying pathology, exercise may trigger sudden cardiac arrest through coronary shear stress, catecholamine surge, hemodynamic overload, hyperthermia, and electrolyte or acid–base disturbance [10,11]. The established link between exercise and SCD from cardiomyopathy, and the evidence that, in certain cardiomyopathies, exercise may promote progression of the underlying disease process, has historically resulted in restrictive exercise recommendations in all affected patients regardless of type of cardiomyopathy, disease severity, presence and burden of symptoms and general risk profile [8,9,12]. As a result, individuals with cardiomyopathy have often been advised to adopt a markedly restricted level of physical activity due to concerns about potential SCD, which may in turn contribute to additional cardiovascular risk as well as adverse psychological effects. In recent years, an increasing body of clinical data has been collected from large cohorts of patients with cardiomyopathies, cohorts that are less selected and therefore more representative of the broad clinical spectrum of these diseases. Analyses of retrospective studies have indicated that physical activity is not necessarily associated with disease progression or an increased risk of sudden cardiac death, although important differences emerge across the various types of cardiomyopathies [2]. Hence, a personalized, risk-stratified approach is essential. Furthermore, compared with earlier evidence, we now also have data concerning sports participation in patients with a prior implantable cardioverter-defibrillator (ICD), either for primary or secondary prevention. Finally, structured rehabilitation programs for the management of heart failure complicating these conditions have been evaluated and proposed. In this review, we aim to provide an updated and comprehensive overview of the current evidence on physical activity—including sports participation and cardiac rehabilitation—in the major cardiomyopathies, and to discuss implications for clinical practice, including shared decision-making, ICD considerations, and genotype-positive/phenotype-negative individuals.

## 2. Hypertrophic Cardiomyopathy (HCM)

HCM is the most common inherited cardiomyopathy and is diagnosed in the presence of unexplained left ventricular hypertrophy not attributable to hemodynamic overload. HCM has a prevalence of 1:200–1:500 in the population, but only 10–20% cases are clinically identified [13]. HCM has heterogeneous clinical presentation and natural history, but pathological substrate is common and is characterized by diastolic dysfunction, left ventricular noncompliance, microvascular abnormalities and remodeling with subendocardial ischemia, hypercontractility, myocardial disarray, aberrant cellular energetics, all conferring to myocardium a high arrhythmogenic potential [2]. This substrate may predispose patients to acute events and physical activity may act as a trigger. Traditionally, guidelines have suggested restrictive policies, discouraging vigorous or competitive sport participation. Indeed, HCM patients are frequently sedentary since they spend less time in recreational physical activity, reducing or interrupting all kinds of sport at the time of diagnosis [14]. Sedentariness is associated with obesity and, in particular in HCM patients, obesity not only increases general cardiovascular risk but also LV mass and burden of HF symptoms [15,16]. However, since HCM is phenotypically heterogeneous, contemporary evidence does not discourage physical activity in HCM patients, but, on the contrary, promotes a more personalized prescription. Indeed, the recent ESC 2023 Guidelines for the Management of Cardiomyopathies [2] and the 2024 AHA/ACC Guideline for the Management of Hypertrophic Cardiomyopathy [17] suggest that low-to moderate-intensity exercise may be beneficial and safe in HCM patients. Indeed, these studies showed improvements in exercise performance, ventilatory efficiency, reduced self-perceived physical limitations and quality of life measures across physical, emotional, and social domains, without a rise in arrhythmias or SCD. They showed an increase in VO_2_max, a reduction in LV filling pressures and of VE/VCO2 slope and an improvement in NYHA class [12,13,15,18,19,20,21,22]. Whereas information about safety of vigorous exercise/high-intensity competitive sport is still limited [2]. A large, multicenter prospective HCM cohort (LIVE-HCM) reported that patients who participated in vigorous or competitive sports did not experience, over 3-year follow-up, higher rates of the prespecified composite end point of death, resuscitated sudden cardiac arrest, arrhythmic syncope, or appropriate ICD therapies compared with non-vigorous participants [23]. In a randomized trial involving 80 adult patients, the safety and effects of high-intensity exercise were compared with usual care. After a 12-week supervised exercise program, participants in the high intensity exercise group demonstrated significantly greater improvements in peak oxygen consumption and VO_2_ at the anaerobic threshold, along with reductions in hospital anxiety and depression scores, compared with those receiving usual care. Importantly, no increase in arrhythmias or adverse event was observed [24]. A retrospective study including 53 athletes with morphologically mild non obstructive HCM, who were largely asymptomatic and carried a low risk profile for sudden cardiac death (SCD), followed their continued participation in competitive sports. Over a mean follow-up of 4.5 years, no deaths, sustained ventricular tachycardia, or syncopal episodes were reported. Importantly, ongoing sport activity did not appear to exert a negative influence on either the morphological or functional expression of the HCM phenotype [25]. These observations corroborate the results of a previous Italian investigation conducted in a smaller cohort [26]. Therefore, the above cited guidelines [2,17] recommend shared decision-making also for participation in vigorous recreational or competitive activities, emphasizing expert HCM evaluation, serial imaging, ambulatory rhythm monitoring, and recognition of high-risk markers (severe wall thickness ≥ 30 mm, prior unexplained syncope, documented sustained VT, family history of premature SCD) that still warrant restriction or consideration of ICD therapy. Regarding LVOT obstruction, most retrospective and prospective studies excluded patients with obstructive physiology, except for one small prospective study not powered for safety and limited to baseline gradients < 50 mmHg [27]. Consequently, there is broad agreement that moderate-to-high-intensity exercise is not recommended in patients with significant LVOT obstruction [2,17]. Symptomatic patients with obstructive HCM (NYHA III–IV) often benefit from surgical myectomy or alcohol septal ablation, whereas mildly symptomatic individuals (NYHA II) are typically treated with beta-blockers, disopyramide, or, more recently, cardiac myosin inhibitors (mavacamten, aficamten), which reduce LVOT gradients and improve exercise tolerance [2,17]. Although no specific data are available, it is reasonable to reconsider physical activity prescriptions—and permissible exercise intensity—in patients who achieve a stable reduction in LVOT gradients through medical or invasive therapy, in light of their newly reassessed clinical and hemodynamic characteristics [28]. It is important to emphasize that exercise prescription in HCM should be individualized—ideally guided by CPET-derived thresholds and tailored to the patient’s clinical profile. A structured FITT (Frequency–Intensity–Time–Type)-based approach has been recently proposed, offering a useful framework for defining frequency, intensity, time, and type of training in this population [29]. Overall, 2023 ESC guidelines on cardiomyopathies, 2024 AHA/ACC HCM guidelines and the 2024 HRS and 2025 AHA/ACC scientific statements recognize that participation in high-intensity exercise or competitive sports may be reasonable for carefully selected patients with a low risk profile, provided that decisions are made through comprehensive expert evaluation and shared decision-making [2,17,30,31]. Nevertheless, because uncertainties remain regarding the long-term outcomes of such participation, ongoing clinical surveillance and individualized counseling are strongly recommended to ensure patient safety [32].

## 3. Dilated Cardiomyopathy (DCM)

**Figure 1:**
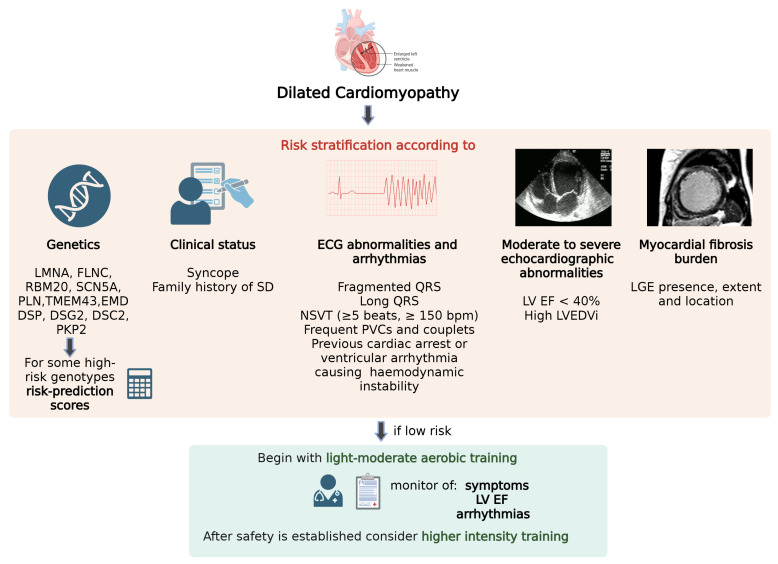
Risk stratification and monitoring required prior to the initiation of physical activity and any subsequent escalation in exercise intensity in DCM.

Dilated cardiomyopathy (DCM) is a disease of the myocardium, not associated with ischemia or valvular disease, in which ventricles become dilated and contractile function is reduced. Patients may have heart failure symptoms, arrhythmias and are at risk of sudden cardiac death. The pathophysiology is heterogeneous: it may be idiopathic, genetic, viral, drugs-related or have other, rarer, etiologies. Mutations associated with DCM predominantly involve genes encoding sarcomeric, cytoskeletal or nuclear envelope [2]. The most common gene related to DCM is titin (TTN) that is found in up to 25% of familial DCM cases, resulting in reduced sarcomere integrity and ventricular dilation [33]. Other genes include lamin A/C (LMNA), linked to a high risk of arrhythmias and sudden cardiac death, filamin C (FLNC), phospholamban (PLN), β-myosin heavy chain (MYH7), cardiac troponin T (TNT2) and desmin (DES) [2]. DCM is characterized by marked heterogeneity, encompassing variations in etiology, left ventricular systolic function, degree of myocardial fibrosis, clinical presentation, arrhythmic profile, and therapeutic response. DCM is the cardiomyopathy with the strongest evidence supporting the benefits of physical activity and cardiac rehabilitation. Most of this evidence, however, originates from studies in patients with heart failure with reduced ejection fraction and has been subsequently extrapolated to DCM, rather than being derived from DCM-specific investigations. Indeed, the 2023 ESC guidelines for the management of cardiomyopathies recommend exercise because it enhances NYHA functional class, cardiopulmonary performance, and overall quality of life [2]. Studies have shown that physical activity leads to better left ventricular function, reductions in end-systolic and sometimes end-diastolic volumes (consistent with reverse remodeling), and significant gains in functional capacity, with VO_2_ peak increasing by 8% to 27%. Quality of life and symptom burden are also favorably affected [34,35,36,37,38,39]. Improvements in functional capacity translate into better outcomes, as higher peak VO_2_ has been associated with a significantly lower risk of all-cause and cardiovascular mortality, as well as reduced rates of all-cause and cardiovascular hospitalization [37]. Regular low- to moderate-intensity exercise is recommended in all DCM patients [2]. On the contrary, symptomatic individuals with DCM are generally advised to avoid competitive sports and recreational activities of moderate to high intensity. Indeed, intensive exercise and competitive sports may precipitate fatal arrhythmias in DCM, particularly in patients carrying mutations associated with intrinsically high arrhythmic risk—such as LMNA, FLNC, transmembrane protein 43 (TMEM43), and PLN [2] or in patients with severely impaired left ventricular function (EF < 40%), complex arrhythmias, or with significant structural alterations such as ventricular dilation and fibrosis [40]. An appropriate risk stratification of the individual patient within expert cardiomyopathy center must precede exercise prescription (Figure 1). High-volume or -intensity exercise could increase wall stress, lead to adverse remodeling, promote fibrosis, or precipitate decompensation if underlying reserve is low. Therefore, the guidelines recommend that high-intensity exercise and competitive sport may be considered only in a select group of asymptomatic (NYHA functional class I), optimally treated individuals, without recent decompensation or history of unexplained syncope, with a left ventricular ejection fraction ≥ 50% and in the absence of exercise-induced complex arrhythmias [2]. In this case, it is suggested to begin with moderate aerobic training; monitor response (symptoms, ejection fraction, arrhythmias) and only after safety is established consider higher intensity training. An ongoing trial will provide valuable insights into the safety and efficacy of personalized exercise training in DCM patients, inform clinical practice and contribute to the development of heart failure management programs [41].

Multiparametric approach to arrhythmic risk stratification including genetics, clinical status, ECG abnormalities and arrhythmias, echocardiographic abnormalities and myocardial fibrosis burden. In low-risk patients it is suggested to begin with light-moderate aerobic training, monitoring symptoms, ejection fraction, arrhythmias and only after safety is established higher intensity training can be considered.

LGE: Late Gadolinium Enhancement; LVEDVi: left ventricular end diastolic index; LVEF: left ventricular ejection fraction; NSVT: nonsustained ventricular tachycardia; PVC: premature ventricular contraction; SD: sudden death. Created with BioRender.

## 4. Arrhythmogenic Cardiomyopathy (ACM)

The broader term of “arrhythmogenic cardiomyopathy”, encompasses all the phenotypic expressions of this cardiomyopathy (predominant RV involvement “classic Arrhythmogenic Right Ventricular Cardiomyopathy”, biventricular involvement, and left-dominant ACM).

**Figure 2:**
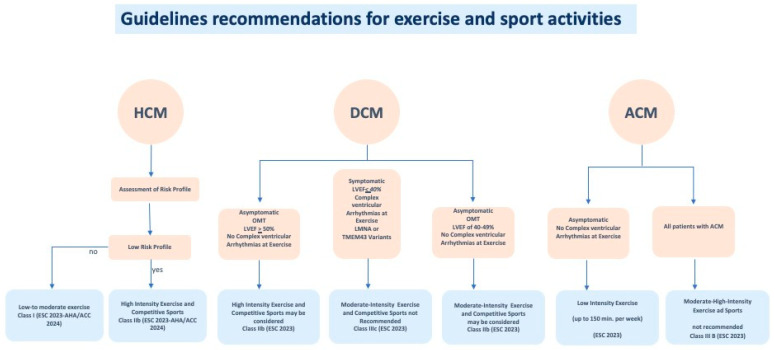
Guidelines recommendations for exercise and sport activities in HCM, DCM and ACM, according to clinical status of the affected individuals.

ACM is an inherited form of heart disease characterized pathologically by fibrofatty myocardial replacement and clinically by prominent ventricular arrhythmias and impairment of ventricular systolic function (biventricular involvement is often observed), thus predisposing to sudden cardiac death, particularly in young patients and athletes [42]. In most cases, ACM is typically caused by an alteration of the intercalated disc, causing disruption of normal cell adhesion and reducing mechanical stability. Indeed, desmosome mutations are the most common cause of ACM and the principal genes involved are plakophillin-2 (PKP2), desmoglein-2 (DSG2), desmoplakin (DSP), desmocollin-2 (DSC2), and plakoglobin (JUP) [42]. The phenotypic expression of ACM is heterogeneous, ranging from genotype positive/phenotype negative patients to symptomatic patients with malignant ventricular arrhythmia, arrhythmic cardiac arrest, advanced heart failure with the necessity of heart transplant [42]. Intense physical exercise is one of the strongest and most consistently demonstrated environmental modifiers of ACM and it influences the disease at multiple levels: increasing age-related penetrance among genetically predisposed individuals, accelerating structural remodeling, and raising the short- and long-term risk of malignant ventricular arrhythmias and sudden cardiac death [43,44,45,46,47], particularly in patients with PKP2 variants [2,48]. Exercise intensity is a particularly strong and independent correlate of life-threatening ventricular arrhythmias in ACM patients: vigorous activities (e.g., competitive running, intense team sports) causes high RV and wall stress [49], accelerating the disease progression [47]. Studies show that vigorous exercise is correlated with greater right ventricular dilatation, reduced right ventricular function and more frequent late gadolinium enhancement (a surrogate of myocardial fibrosis) both in mutation carriers and affected patients [47]. Repeated high wall stress is thought to exaggerate mechanical injury of myocytes whose desmosomal integrity is already compromised, promoting myocyte loss, inflammation and fibrofatty replacement that create the substrate for reentrant ventricular arrhythmias. In addition, intense exercise has shown to induce different modifications depending on the genotype [50]. In non-familial forms of ACM, intensive exercise has been proposed to play a disproportionate role in pathogenesis by altering cardiac structure and promoting arrhythmogenesis and increasing the likelihood of ventricular arrhythmias [51]. For this reason, exercise restriction is a core component of management for patients with definite ACM and for genotype-positive individuals; moderate- and/or high-intensity exercise, including competitive sport, is not recommended in individuals with ACM [2]. However, while many cohort studies support exercise restriction, randomized controlled trials are lacking, and existing data are observational or retrospective so the magnitude of benefit from sports restriction are inferred rather than proven. Several clinically relevant doubts remain. First, lower-intensity recreational activity appears to carry substantially less risk and provides cardiovascular and psychosocial benefits; advising complete sedentary isolation is neither necessary nor desirable for most patients [2,46]. Secondly, inactivity leads to increased morbidity and mortality. Observational evidence suggests that reducing exercise after diagnosis has been shown to improve clinical outcomes in patients with ACM [47,49], with lower incidence of subsequent ventricular arrhythmias and appropriate ICD therapies [49]. Notably, the intensity of the exercise rather than the duration was correlated with the worst outcome [44]. For this reason, it has been hypothesized that low–moderate-intensity exercise training may have beneficial effects by reducing cardiovascular risk without accelerating the disease progression [46,52]. Although no universally accepted thresholds exist, large, international cohorts and registries have confirmed a dose–response relationship: lower exercise exposure (approximately ≤10–15 MET-hours/week) is generally associated with a more favorable safety profile, whereas higher volumes (roughly >15–30 MET-hours/week) have been linked to meaningful incremental risk, especially in females [44,47,49,50,52,53]. These values should be interpreted as approximate reference ranges rather than prescriptive cut-offs and individualized within a comprehensive clinical evaluation.” In practical terms, clinicians should identify genetic status, evaluate RV and LV structure/function and fibrosis with echocardiogram and CMR, arrhythmic burden with Holter ECG and exercise testing, and then provide a personalized approach based on the principle that genotype-positive/phenotype-positive patients with high-risk features should avoid high-intensity and competitive endurance sports; while lower-risk patients may consider low–moderate activity with periodic re-evaluation or if new symptoms occur [50,53]. Therefore, physicians should adopt a cautious yet individualized approach that prioritizes patient safety while acknowledging the potential harms of unnecessary lifelong restriction from physical activity (Figure 2 and Table 1).

Low risk profile: asymptomatic, low-risk HCM individuals with morphologically mild HCM in the absence of resting or inducible left ventricular outflow obstruction and exercise-induced complex ventricular arrhythmias; OMT: optimal medical therapy. The overall intensity of physical activity can be quantified using weekly metabolic equivalents (MET-h/week), classified approximately as follows: <10 MET-h/week for light activity, 10–15 MET-h/week for moderate activity, and >15–30 MET-h/week for vigorous activity, with values > 30 MET-h/week representing very high exercise volume.

## 5. Implantable Cardioverter Defibrillator and Sport Activity

Implantable cardioverter-defibrillators (ICDs) remain the cornerstone of sudden cardiac death (SCD) prevention in patients with cardiomyopathies, yet their role in athletes continues to be debated. While ICDs are highly effective in terminating malignant ventricular arrhythmias, longstanding concerns have focused on the safety of competitive sports, the potential for device-related complications, and the psychological impact of shocks. Historically, individuals with genetic heart diseases predisposing to SCD have been broadly restricted from competitive athletics. However, over the past decade, registry-based and prospective studies have begun to reshape this paradigm, supporting a more nuanced approach that weighs arrhythmic risk against the recognized benefits of physical activity. Emerging data on return-to-play (RTP) within shared decision-making (SDM) frameworks indicate that event rates in athletes with genetic heart disease are lower than previously assumed, and the consideration of ICD implantation for primary prevention in athletes—taking into account their athletic status—has emerged as a clinical possibility. The first meaningful evidence came from a registry published in 2013, which included 372 ICD recipients, 60 of whom continued competitive sports such as running and soccer [54]. During a median follow-up of 31 months, no deaths or device failures were observed, although 18% of athletes experienced shocks, both appropriate and inappropriate. Predictors of appropriate shocks included younger age and conditions such as arrhythmogenic cardiomyopathy, catecholaminergic polymorphic ventricular tachycardia (CPVT), and idiopathic ventricular fibrillation, highlighting the heterogeneity of risk among subgroups [54]. Further reassurance came from the prospective ICD Sports Registry, which followed 440 athletes aged 10–60 years, nearly half with a prior history of ventricular arrhythmias [55]. The most common underlying diagnoses were long QT syndrome, hypertrophic cardiomyopathy, and ARVC. No failures of defibrillation, deaths, or injuries due to arrhythmic syncope were reported. About 10% of participants received shocks during practice or competition, corresponding to an annual incidence of 3%. Although shocks occurred more often during physical activity than at rest, no significant differences emerged between competitive and recreational exercise [55]. A European study reported a relatively low 10-year risk of lead malfunction among athletes who continued sporting activity after transvenous ICD implantation [56]. A recent study from the Mayo Clinic followed 125 athletes with genetic heart diseases at risk for arrhythmic SCD—including LQTS, HCM, and ACM—who had an ICD implanted and were formally cleared for return to play [57]. During a mean follow-up of 3.6 years, these athletes experienced an annual arrhythmic event rate of 6.3%, substantially higher than the 0.3% per year observed in a larger cohort of 533 athletes with genetic heart disease but without ICDs. Notably, however, ICD shocks occurred with equal frequency during sports and outside of sports, reinforcing the view that those who required VT/VF-terminating therapy had been appropriately risk-stratified and implanted for rigorous clinical reasons. Rates of inappropriate shocks and device-related complications were relatively low, at 1.34% and 5% per year, respectively [57]. Collectively, these findings demonstrate that ICDs are effective in terminating ventricular arrhythmias both in recreational and competitive sports and suggest that selected athletes with ICDs can safely participate in vigorous or competitive exercise without excessive risk of device failure, inappropriate therapy, or injury. In this context, tailored device programming is essential. In athletes, high-rate cutoffs are associated with fewer overall and inappropriate shocks, both during competition and training, while prolonged detection intervals further reduce total shocks, alleviating psychological distress without compromising safety [58]. To optimize device programming, exercise stress testing can be used to identify the maximal sinus rate an individual is likely to achieve during physical activity, ensuring settings that are both protective and safe. In addition, a post-implantation recovery period should be completed before consideration of resumption of competitive sport participation [30].

Nevertheless, disease-specific considerations are crucial. In ACM, one of the most challenging contexts for return-to-play, exercise has consistently been associated with acceleration of phenotypic expression and adverse outcomes, leading to long-standing restrictions in guidelines. Participation in high intensity or frequent endurance exercise is not recommended, particularly for patients with ACM, due to an association with increased risk of ventricular arrhythmias and sudden death. In one study of these patients, competitive sport was associated with two-fold increased risk of ventricular arrhythmias, death, and symptoms compared with patients who were inactive or who participated in recreational sports [31]. Although ICDs remain effective in terminating arrhythmias even during exercise, small registries suggest a slightly higher rate of breakthrough cardiac events in ACM athletes, though the evidence remains underpowered. Whether athletes with ACM should be considered for a structured shared decision-making (SDM) process is still debated and will require data from larger cohorts.

In DCM, physical activity may contribute to arrhythmogenesis and accelerate disease progression. Current recommendations advise that patients with left ventricular ejection fraction ≤ 40%, exercise-induced arrhythmias, or pathogenic variants in LMNA, TMEM43, or filamin C refrain from vigorous exercise or competitive sports, even if protected by an ICD [2,31].

By contrast, in HCM, ICDs are generally effective in preventing sudden cardiac death, although defibrillation thresholds are often higher in this population, making defibrillation testing particularly important. In patients with HCM, participation in vigorous exercise was not associated with an increased risk of death, cardiac arrest, or ICD shocks compared with engagement in low- to moderate-intensity activity [23]. Data from the ICD Sports Registry further showed that although ICD shocks were relatively frequent among competitive athletes with HCM, life-threatening ventricular arrhythmias were uncommon and consistently terminated by the first shock [55,59].

Another important area of uncertainty relates to subcutaneous ICDs (S-ICDs). All available data on sports safety to date pertain to standard transvenous systems. Because of the course of the subcutaneous lead beneath major chest muscles, theoretical concerns exist regarding fracture or damage during sports involving chest or shoulder strain, such as weightlifting or swimming, and even in low-impact sports like golf. Moreover, the extrathoracic position of the lead may increase susceptibility to trauma from collisions or projectiles. To address these questions, the SPORT S-ICD trial, a multicenter observational study enrolling 450 patients with S-ICDs who continue regular or competitive exercise is underway. Its findings will be critical for shaping recommendations for this growing patient population [60].

Finally, the psychosocial dimension cannot be overlooked. Athletes who receive shocks, whether appropriate or inappropriate, consistently report reduced quality of life, with heightened anxiety for both patients and families. While some athletes withdraw from sport after shocks, the majority eventually resume exercise, reflecting the considerable psychological and physical benefits derived from sports participation.

**Figure 3:**
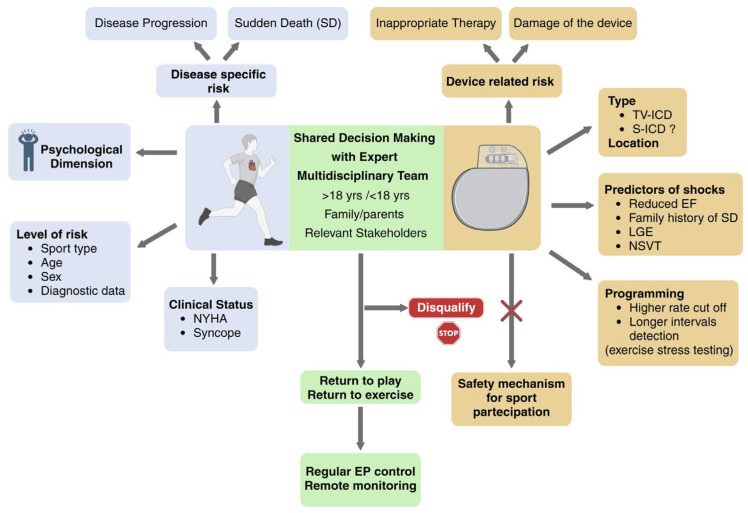
Implantable Cardioverter-Defibrillator and Sports Participation in Patients With Cardiomyopathy: The Central Role of Shared Decision-Making. TV-ICD: transvenous ICD; S-ICD: subcutaneous ICD; LGE: late gadolinium enhancement at cardiac magnetic resonance; NSVT: non-sustained ventricular tachycardia. Created with BioRender.

*Shared decision-making* (SDM). Emerging evidence and the growing integration of shared decision-making into clinical practice have contributed to a substantial evolution of guidelines and a paradigm shift in the management of athletes with cardiovascular disease who wish to return to play (RTP). The goal of SDM is to provide athletes with comprehensive information about the potential risks—including sudden cardiac death—and benefits of sports participation, while incorporating their values and preferences into the decision-making process. Risk stratification is individualized, taking into account sport type, age, sex, and diagnostic findings. Some athletes, after understanding their risk profile, may choose to self-disqualify, whereas others may elect to continue despite medical recommendations [61]. Importantly, SDM should involve not only the athlete but also their family, team, and all relevant stakeholders. When the patient is younger than 18 years, the discussion must be adapted to ensure decisions are shared between the pediatric patient and their parents, reflecting their collective values and priorities. Moreover, SDM must remain disease- and patient-specific, given that each cardiomyopathic substrate carries distinct arrhythmic and device-related risks. Finally, current international cardiology guidelines strongly caution against viewing ICD implantation as a ‘safety mechanism’ to justify sports participation, emphasizing that an ICD should never be implanted solely to permit competitive activity and its presence cannot be considered a safeguard that mitigates the intrinsic arrhythmic risk associated with high-intensity exercise [2,17,31]. In conclusion, contemporary data suggest that ICDs generally perform reliably in athletes with cardiomyopathies, but disease-specific risk remains an essential determinant of RTP decisions. A universal prohibition of sports is not supported; rather, an individualized, shared decision-making approach, ideally within expert multidisciplinary teams, represents the most balanced path forward (Figure 3).

## 6. Sports Participation in Genotype-Positive/Phenotype-Negative Athletes with Genetic Cardiomyopathies

The 2025 American Heart Association and American College of Cardiology (AHA/ACC) Scientific Statement on Competitive Sports Participation for Athletes with Cardiovascular Abnormalities emphasizes that a uniform mandate of sports restriction across all genetic cardiomyopathies should not be applied [30]. Instead, competitive sports participation may be reasonable in selected athletes with genetic cardiomyopathies following comprehensive evaluation and shared decision-making [30]. Genotype-positive/phenotype-negative (G+/P−) individuals carry a pathogenic or likely pathogenic variant without manifesting clinical features of the disease. The growing use of genetic testing has increased their identification, raising challenges in clinical management. Because variant expressivity is highly variable and phenotypic conversion is unpredictable, the role of environmental factors—particularly exercise intensity remains uncertain [62]. Presently, the absence of validated risk models and prospective data, along with limited long-term follow-up and quantification of exercise exposure, hampers accurate SCD risk assessment in genotype-positive, phenotype-negative individuals. Current sports participation guidelines recognize these knowledge gaps, and recommendations for engaging in intensive or competitive activity remain cautious and individualized. Therefore, participation decisions should be individualized and made jointly with the athlete and their family, taking into account the family history of SCD, type and intensity of sport, and the athlete’s personal risk tolerance [30].

**DCM**. In G+/P− individuals, participation in intensive or competitive sports may generally be permitted in the absence of overt left ventricular dysfunction or dilation [2]. However, special caution is warranted in carriers of LMNA or TMEM43 variants, as emerging evidence suggests that exercise may exacerbate myocardial dysfunction and increase the risk of life-threatening arrhythmias [2]. For other gene carriers without phenotypic expression, moderate- to high-intensity physical activity can be considered, provided that individualized evaluation and longitudinal follow-up are ensured [2].

**ACM**. In G+/P− athletes, genotype-informed discussions are recommended to address the potential relationship between high-intensity endurance exercise and the development of overt ACM phenotype or ventricular arrhythmias [31]. According to the 2023 ESC Guidelines, avoidance of high-intensity or competitive exercise may be considered for G+/P− carriers, particularly within families with ACM or in carriers of ACM-related pathogenic variants [2,63]. Participation in moderate-intensity activities can, however, be considered when individualized assessment suggests low arrhythmic risk.

**HCM**. G+/P− individuals in HCM represent a unique clinical group, increasingly recognized due to widespread use of genetic testing. These subjects carry pathogenic sarcomeric mutations but do not present evidence of the disease on imaging. The management of physical activity in this population is particularly challenging, as the risk of SD appears extremely low but is not negligible. Current guidelines suggest that G+/P− individuals can safely participate in vigorous and even competitive sports, provided that they undergo periodic re-evaluation [2,17]. Longitudinal studies have shown very low rates of conversion to overt HCM during follow-up, and adverse cardiac events are rare [17,26,63,64]. Similar findings have also been observed in animal models [65]. International guidelines emphasize individualized counseling and periodical monitoring with electrocardiography (ECG), echocardiography, cardiac magnetic resonance and ECG Holter analysis [17,32]. Unlike patients with overt HCM, G+/P− individuals are generally not restricted from intense exercise, as training itself does not appear to promote disease expression or progression [62,65]. Nonetheless, shared decision-making is crucial, particularly in families with a history of SD or malignant arrhythmias. Therefore, evidence supports liberal exercise prescription in G+/P− subjects, while continuing structured surveillance to promptly detect phenotypic conversion or arrhythmic manifestations. In summary, contemporary evidence supports a personalized, genotype- and phenotype-informed approach to sports participation in athletes with genetic cardiomyopathies. Blanket restrictions are no longer justified; instead, recommendations should integrate genetic profile, disease expression, sport type and intensity, family history, and shared decision-making among the athlete, family, and multidisciplinary care team (Table 2).

## 7. Exercise Prescription and Rehabilitation in Cardiomyopathies

Cardiac rehabilitation (CR) is a comprehensive, multidisciplinary intervention that encompasses physician-prescribed exercise training (ET), risk factor modification, psychosocial support, nutritional guidance, weight management, and systematic evaluation of clinical outcomes, all aimed at improving prognosis. Robust evidence supports the beneficial impact of CR programs across a wide range of cardiovascular diseases. Among their core components, ET has consistently demonstrated the capacity to enhance exercise tolerance, improve lipid profiles, alleviate symptoms, and reduce mortality. Despite its established benefits, cardiac rehabilitation continues to be underused in clinical practice. This is particularly evident in the field of cardiomyopathies, where implementation remains sporadic and the existing evidence comes almost exclusively from a few specialized centers.

**HCM**. Contemporary European and North American guidelines on HCM highlight the benefits of structured, supervised exercise programs as part of comprehensive management [2,17]. Despite increasing evidence supporting their safety, participation in CR among HCM patients remains low, mainly due to concerns about exercise-induced ventricular arrhythmias and sudden cardiac death (SCD). As a result, many patients are discouraged from engaging in physical activity and tend to lead sedentary lives, which contributes to reduced cardiorespiratory fitness, diminished functional capacity, and lower bone and muscle health. However, recent studies suggest that the risk of SCD associated with exercise in individuals with HCM may be lower than historically reported [17]. Moreover, clinical studies of cardiac rehabilitation in older HCM patients have demonstrated the safety and physiological benefits of supervised exercise when performed within appropriate limits [21,22,66,67]. A recent systematic review evaluated studies incorporating exercise training as part of CR programs in HCM to assess their impact on patient outcomes. Five studies, published between 2013 and 2023 and including a total of 235 participants, were analyzed [68]. The main findings indicate that expert-supervised CR is safe and results in significant improvements in functional capacity (peak VO_2_ and METs, up to +43%), reductions in body weight and body mass index (BMI), optimization of blood pressure, and improvements in echocardiographic parameters such as left atrial diameter and pulmonary artery systolic pressure, along with other cardiometabolic markers. No major adverse events—including sudden cardiac death or sustained arrhythmias—were reported; a few studies documented non-sustained ventricular tachycardia episodes during exercise. Nevertheless, further research is needed to compare different exercise modalities and to develop individualized, disease-specific training protocols for patients with HCM.

**DCM**. Exercise prescription is among the most effective interventions for improving cardiopulmonary function, NYHA functional class, and quality of life, and is therefore recommended by current ESC heart failure guidelines [2]. Although DCM is the cardiomyopathy with a strong evidence base for exercise intervention, much of this evidence is extrapolated from studies in patients with heart failure with reduced ejection fraction and is not specific to DCM [6]. Disease-specific data remain limited and are generally derived from small study populations. Nonetheless, several investigations have demonstrated the efficacy and safety of exercise-based CR in DCM, with consistent improvements in functional capacity, though its application in advanced stages of disease remains limited. The largest study to date, conducted by Mehani [35], was a randomized controlled trial including 40 patients, of whom 30 completed the study (15 in the exercise group and 15 in the control group). Participants underwent baseline and post-intervention evaluation with cardiopulmonary exercise testing (CPET), echocardiography, and the Kansas City Cardiomyopathy Questionnaire (KCCQ). The exercise group completed a seven-month supervised aerobic training program, starting at 55% of maximum heart rate and progressing to 80% by the end of the period. Only the training group demonstrated a significant increase in peak VO_2_ (from 16.1 ± 3.65 to 21.08 ± 5.47 mL/kg/min), accompanied by a reduction in resting heart rate, improved diastolic filling, and higher quality-of-life scores on the KCCQ. A smaller study assessed the feasibility and potential benefits of exercise-based CR in 18 patients with biopsy-proven inflammatory cardiomyopathy (ICM), matched for age, sex, left ventricular ejection fraction, and baseline in-hospital therapy, including renin–angiotensin–aldosterone system inhibitors, cardiac resynchronization therapy (CRT-D), and immunosuppression for virus-negative myocarditis [66]. CR led to faster and greater recovery of LVEF compared with controls (3 months vs. 13 months; 42% vs. 31%, respectively), along with a significant reduction in NT-proBNP levels. Importantly, CR appeared to be safe and potentially beneficial even during the active phase of myocarditis [69]. More recently, a case report described a 57-year-old man with idiopathic DCM and severe systolic dysfunction (LVEF = 5%) characterized by inferoseptal fibrosis and biventricular dilation on cardiac magnetic resonance [70]. The patient, on optimal medical therapy, underwent a supervised program of 30 min aerobic training sessions twice weekly for a total of 24 sessions. Marked functional improvement was observed, with a 114% increase in the 6 min walk distance (from 280 m [35% of predicted] to 600 m [82% of predicted]), an increase in metabolic equivalents on stress testing (from 7.0 to 7.8), and a substantial improvement in heart rate recovery (from 13 to 27 beats in the first minute).

**ACM**. Regular exercise provides well-established benefits for cardiovascular and overall health. However, in arrhythmogenic cardiomyopathy (ACM), high-intensity or endurance exercise may accelerate disease expression and increase susceptibility to malignant ventricular arrhythmias. Experimental and clinical studies in both human and animal models consistently support a causal link between vigorous exercise and adverse remodeling in ACM. At present, no clinical data are available on the efficacy or safety of structured exercise or cardiac rehabilitation (CR) programs in ACM, and existing evidence is limited, genotype-specific, and mainly retrospective. Current knowledge supports an individualized, cautious approach, emphasizing tailored clinical evaluation, low- to moderate-intensity exercise, and annual follow-up to monitor potential disease progression in athletes or active patients who wish to continue exercising. Cardiopulmonary exercise testing should guide exercise prescription by identifying ventilatory thresholds and determining safe intensity levels [50]. Training programs must be supervised by professionals with expertise in inherited cardiac diseases and ideally conducted in specialized centers of excellence. In conclusion, although in ACM patients a low- to moderate-intensity physical activity appears reasonable and beneficial for overall well-being, evidence on its long-term safety and impact on disease progression remains limited, underscoring the need for prospective studies to inform future recommendations and the applicability of exercise training and rehabilitation programs in these patients.

## 8. Discussion

Physical exercise and sports participation are no longer a taboo for patients with cardiomyopathy. Over recent years, the accumulation of large clinical datasets and broad patient cohorts—representing the full clinical spectrum of these conditions—has reshaped our understanding of this topic. From early single-center experiences to retrospective analyses and, more recently, prospective trials, the traditional paradigm of strict exercise restriction has been progressively challenged. It has become evident that the impact of exercise on cardiomyopathies is not uniform. Its effects on disease progression, structural remodeling, and clinical or prognostic outcomes vary according to the specific cardiomyopathy subtype, its underlying pathophysiology (preserved vs. reduced systolic function), pathogenic mechanisms (hypertrophic remodeling versus structural weakening), genetic substrate, and intrinsic risk of sudden cardiac death. The work, however, is far from complete. Many aspects remain to be clarified—particularly the mechanisms and magnitude of exercise effects across different cardiomyopathies, as well as the development of optimal and standardized exercise protocols for clinical implementation. Evidence guiding exercise and sports participation in children and adolescents with cardiomyopathies remains limited, as most available data derive from adult or late-adolescent cohorts. Risk stratification in younger patients is inherently challenging: existing pediatric-specific models rely on retrospective multicenter data, which carry limitations such as missing information and potential selection bias [2,71]. Many cardiomyopathies show age-dependent penetrance, so young patients may not yet have a fully developed phenotype at first evaluation. This underscores the need for regular re-assessment (at least annually) to monitor clinical status, arrhythmias, and structural evolution. Data on the safety of high-intensity exercise in youth are scarce, and decisions about competitive sports should therefore be individualized and based on age-appropriate risk factors. Most children with cardiomyopathy can participate in school physical education with appropriate adaptations. Overall, more refined pediatric risk stratification tools and prospective data are needed to inform safe exercise recommendations across the spectrum of cardiomyopathies in this age group.

Nonetheless, when faced with patients with cardiomyopathy wishing to engage in physical activity or competitive sports, clinicians can now rely on increasingly refined tools to assess individual risks and benefits. This complex evaluation requires in-depth knowledge of each specific disease—its natural history, from the genotype-positive/phenotype-negative stage to advanced phases, and its expected trajectory according to the most recent evidence—and should be undertaken by cardiologists with specific expertise in the management of cardiomyopathies. The physician–patient interaction plays a pivotal role within a structured shared decision-making process, which has become an essential step toward safe and informed participation in exercise and sports. The growing acceptance of physical activity among patients with cardiomyopathies has already shown encouraging short-term benefits in quality of life and cardiovascular health. Whether these improvements will ultimately translate into better long-term outcomes remains to be confirmed by future studies. The care of patients with cardiomyopathy may soon move from seeking medical clearance for exercise to cardiologists proactively prescribing tailored physical activity as part of comprehensive disease management.

## Figures and Tables

**Table 1:** Exercise intensity levels and corresponding sports activities.

Low-Intensity Activities	Moderate-Intensity Activities	High-Intensity/Competitive Sports
Yoga	Recreational swimming	Competitive running
Pilates	Light road cycling	CrossFit
Walking (leisure pace)	Hiking	Triathlon
Stretching	Dance fitness (light Zumba)	Soccer/Football
Golf	Skating	Basketball
Gentle gymnastics	Aerobics	Rugby
Bowling	Amateur tennis	Boxing/Martial arts (competitive)
Leisure cycling	Recreational rowing	Sprinting/Track and field
Low swimming	Social dancing	Competitive tennis
Gentle cycling on flat terrain	Cross-country skiing (moderate)	Competitive swimming

**Table 2:** Sports eligibility and recommendations for Genotype-positive/Phenotype-negative individuals with genetic cardiomyopathies.

Condition	Genetic Variant/Subgroup	Exercise Recommendation	Evidence Level/Class	Primary Source
**Arrhythmogenic Cardiomyopathy (ACM)**	Genotype-positive, phenotype-negative	Discuss potential risk of phenotype expression and arrhythmias with high-intensity endurance exercise; moderate exercise may be considered.	IIb C	Consensus Statement 2024 [54]
	All ACM patients	Avoid high-intensity and competitive sports.	IIb C	ESC 2023 [2]; ESC VA/SCD 2022 [59]
**Hypertrophic Cardiomyopathy (HCM)**	Genotype-positive, phenotype-negative	Competitive sports of any intensity may be reasonable; annual expert reassessment is recommended to monitor phenotype development.	IIa B	AHA/ACC 2024 [17]; ESC 2023 [2]
	Without family history of SCD, risk factors for SCD, LVOTO, malignant arrhythmias	No restriction; routine ambulatory monitoring not required unless indicated.	IIa B	AHA/ACC 2024 [17]
**Dilated Cardiomyopathy (DCM)**	Genotype-positive, phenotype-negative (general)	Intensive or competitive sports generally permitted in absence of LV dilation or dysfunction.	IIa C	ESC 2023 [2]
	Carriers of LMNA or TMEM43 variants	Exercise may worsen function and increase arrhythmic risk; avoid high-intensity and competitive sports.	IIb C	ESC 2023 [2]
	Carriers of other variants (non-LMNA/TMEM43)	Moderate- to high-intensity physical activity may be considered with individualized assessment and follow-up.	IIb C	ESC 2023 [2]
**General Principle**	All genotype-positive, phenotype-negative cardiomyopathies	Uniform restrictions should not be applied; decisions based on genotype, family history, sport intensity, and shared decision-making (SDM).	Consensus	AHA/ACC Scientific Statement 2025 [53]
